# Family Food Behaviours and Adolescents’ Overweight Status: A Mother-Offspring Link Study

**Published:** 2011-11-01

**Authors:** S Babajafari, G C Marks, A A Mamun, M J O’Callaghan, J M Najman

**Affiliations:** 1 School of Health and Nutrition, Shiraz University of Medical Science, Shiraz, Iran; 2 The University of Queensland, School of Population Health, Qld 4006, Australia; 3 Mater Children’s Hospital, The University of Queensland, Brisbane, QLD, Australia

**Keywords:** Family eating, Food behavior, Food choice, Adolescence, Overweight

## Abstract

**Background:**

The role of family food behaviours on weight status of family members is not well understood. The objective of this study was to examine the influence of some aspects of family food behaviours on adolescents overweight.

**Method:**

In a cross-sectional mother-child-linked study design, participants were a subsample of 3862 adolescents (51.9% boys) from the Mater hospital-University of Queensland Study of Pregnancy (MUSP), a longitudinal study of a birth cohort of 7,223 women and their offspring. Information on aspects of family food behaviours (family meal patterns and influences, frequency of family food consumption, and influences on family food selection) was collected by self reported questionnaires from mothers in a 14 years follow up (in 1994-1997) and other covariates at different stages of follow-ups. Body Mass Index of the adolescents was calculated using measured height and weight.

**Results:**

Being overweight at 14 years had significant negative associations with mothers’ report on the importance of family meals (OR=1.28), frequency of eating cake/biscuits (OR=1.71), and significant positive association with mothers’ report on frequency of consumption of cooked vegetables (OR=1.32), soft drinks (OR=1.60) and importance of fattening of foods (OR=1.27).

**Conclusion:**

The study confirmed the importance of the family and some family food behaviours in shaping risk of being overweight amongst adolescents. Because of the cross-sectional nature of this study, we could not conclude that they had casual correlations. Comparison with the literature suggests that some of these effects may be specific to particular contexts, potentially affected by cultural and socio-economic differences.

## Introduction

The rise in prevalence of obesity among children in western societies, including Australia, during the last three decades is well documented.[1][2][3][4] Continuity of overweight and obesity from childhood to adulthood is demonstrated in some studies.[2][5][6] It is usually difficult to reduce weight once obesity is established.[3][7][8] As a result, prevention of the obesity as early as possible is recommended. The recent dramatic increase in prevalence of obesity has led to environmental factors receiving more attention in the etiology of the obesity.[4] These include influences at individual, social, physical and macro-system levels that are assumed to increase the risk of obesity due to their influence on adolescents’ food choice and level of physical activity.

Research has shown that there is clustering of family food patterns and children’s eating patterns.[9] Parent eating styles and preferences appear to have a crucial effect on food choice and obesity risk of adolescents. [10] Daughters of parents who used more fruits and vegetables tended to use more fruits and vegetables. [11] In addition, adolescents prefer to eat foods which were available and readily accessible to them, usually provided by family. Although findings are inconsistent, eating styles and food preference of families, and consequent adolescent’s obesity, could be partly a result of an interaction between a number of factors including taste and cost of foods, convenience, preferences of family members, and concern about healthiness or fattening nature of foods. Evidence regarding the role of likes or dislikes by members of a family for a particular food on the frequency of the consumption of that food is also equivocal.

Despite progress in the past decade in understanding some aspects of the role of the family environment on obesity, evidence regarding the role of psychosocial influences of family meals during adolescence on obesity of adolescents is unclear.[12] Although an association of consumption of sugar sweeteneddrinks with child obesity has been shown in a few studies,[13][14][15] there is limited evidence for the impact of behavioural influences of the family, including meal pattern, on adolescents’ overweight/obesity.[10] Newby et al. (2007) in his recent comprehensive review of the literature on the relation of childhood obesity with dietary intake and eating behaviour, pointed out that there were few studies assessing the effect of frequency of the family eating meals together on adolescent’s body mass index (BMI) and overweight/obesity and the results are not conclusive.

Story et al. (2002) presented a theoretical model of factors that influenced adolescent’s food choice which was used as a guide in our study.[16] The influence of family, as a part of social environment, in food choice of the youth was highlighted in this framework. Psychosocial and behavioural influences and life styles factors in a family can have impacts on children’s food choices. The family can form food choices of members through modelling the food consumption, making certain foods available and by encouraging or restricting selected foods to members.

The available body of literature is not consistent in showing the association of different aspects of family food behaviours with overweight/obesity of adolescents. Most of the findings are inconclusive because of methodological limitations; whether studies were small scale, height and weight were self reported,

conducted in special populations or were not adjusted for potential confounders. Our study is an extension of the study conducted by Mamun et al. (2005) which examined the cross sectional association of mother’s attitude towards whether the family ate together and maternal report of how often they ate together on the odds of the adolescent being overweight. We examine the cross sectional association of a range of social environment factors, including psychosocial, behavioural and life style factors with adolescent’s weight status.

## Materials and Methods

Participants were from the Mater-University of Queensland Study of Pregnancy (MUSP), a longitudinal study of a birth cohort of 7,223 women and their offspring. They received their antenatal care at a major public hospital (Mater Misericordiae Hospital) in South Brisbane, Australia, between 1981 and 1984 and delivered a live singleton baby who was not adopted before leaving the hospital, and completed both initial phases of data collection.[17] The mothers and children were followed-up (FU) prospectively, with maternal questionnaires being administered when their children were 6 months, 5 years, and 14 years. At 5 and 14 years FU, detailed physical, cognitive and developmental examinations of the children and young adults were undertaken. Furthermore, at 14 years FU, the children and young adults completed health, welfare, and lifestyle questionnaires. Participants gave signed informed consent for their participation and that of their children till 5 years, after which individual consent was obtained from the youth. The study protocol conformed to the ethical guidelines of the 1975 Declaration of Helsinki as reflected in a priori approval by the institution's human research committee. Of the original 7223 participants, data on BMI were available for 3862 (53%) adolescent participants (51.9% boys, 48.1% girls) at 14 years FU conducted in 1994-1997. The age range of the participants was 12-15 years. Body Mass Index (BMI=weight in kilograms divided by height in meters squared - Kg/m^2^) of the adolescents was calculated, using their height measured by a portable stadiometer to the nearest cm, and young adult weight measured in light clothing by the average of two measurements with a scale accurate to 0.2 kg. In this study, we used the definition of Cole et al. for overweight for children, for instance for 14 year old adolescent, overweight was defined as a BMI over 22.62 Kg/m^2^ for males and 23.34 Kg/m^2^ for females, which is equivalent to BMI of 25.0 and over for adult.[18] These BMI cut-off points are also equivalent to the 85th percentile on the CDC 2000 scale.[19][20] As the number of participants in the obese group were few in this study, the outcome categories were collapsed into two categories; normal weight and over weight, for example at 14 years of age, normal weight (BMI < 22.62 Kg/m^2^ for males and 23.34 Kg/m^2^ for females) and overweight/obese (BMI ≥ 22.62 Kg/m^2^ for males and BMI ≥ 23.34 Kg/m^2^ for females).

The main exposures for this study were different aspects of family food behaviours reported by mothers at the 14 years FU. We divided the questions reported by the mothers into 3 groups according to Story’s model: Psychosocial influences, behavioural influences and life-style factors:

(i) Psychosocial influences: we examined family meal patterns and influences as a proxy for family’s psychosocial influences on adolescence overweight by analysing mothers’ response to 5 questions:

1) How often does your family sit down to eat a meal together? (once a day, few times a week, once a week, less than once a week )

2) How important to you is it that your family eats together? (not really important, quite important, very important)

3) How often does your family go out to eat? (once a week, once or twice a month, several times a year, rarely or never)

4) How often does your family get take-away foods? (Once or more a week, few times a month, less than once a month, rarely or never).

5) Who decides what food gets bought? (I decide completely, I decide mostly, my partner and I decide equally, my partner decides mostly, my partner decides completely).

The last two categories of the following items were merged during analysis because of small numbers: Maternal report on frequency of family eats together, family going out to eat and family getting take-away food. The last 3 categories of the question “who decides what food gets bought?” also were merged because of small numbers.

(ii) Behavioural influences: We analysed frequency of family food consumption as a proxy for behavioural influences of family on overweight of adolescents by the response to 9 questions asked of mothers:

How often do you have the following? (With the responses categories for each of the 9 questions: Once or more a day, most days, 2 or 3 times a week, rarely or never): Sweets/lollies, fresh fruit, fast food (pizza, burgers, chips), cooked vegetables, salad, red meat, soft drink/cordial, fruit juice and cakes/biscuits.

The following were merged during analysis because of small numbers: The first two categories of sweets, soft drink, fruit juice and cake/biscuit; the first three categories of fast food; the last two categories of fresh fruit, cooked vegetables and salad; the first and last two categories of red meat.

(iii) Life-style factors: we analysed influences on family food selection as a proxy for the influence of lifestyle factors of the family on adolescent overweight by response to 7 questions asked of mothers:

How important were the following when you chose food? (With the response categories for each of the 7 questions: Not important, quite important, very important): Taste of the food, whether my children would eat it, whether my partner would eat it, whether it was easy to prepare, healthy, fattening, and expensive.

The first two categories of the following items were merged during analysis because of small numbers: Taste of the food, whether my children would eat it, whether my partner would eat it, whether it was healthy and whether it was expensive.

A set of variables were identified as potential confounders in our study based on prior knowledge from the literature.[4][16][21][22][23][24][25][26][27] Those variables based on obstetric records or reported by mothers during pregnancy, or around the time of birth of the child were sex, ethnicity (White, Asian, and Aboriginal-Islander), maternal parity (1, 2, 3 or more), maternal BMI (height was measured and pre-pregnancy weight was selfreported; BMI was categorized to normal weight, overweight/obese according to WHO standards), maternal education at birth (Did not complete secondary school, completed secondary school, completed further/ higher education). Other potential confounders reported by mother at 14-years FU were: Gross family income (low, <Au $15599; medium, Au $15600- 31148; and high, Au $31149 or more), amount of time the child spent watching TV (5 hours or more per day, 3 to <5 hours per day, 1 to <3 hours per day, and <1 hours per day); and time spent on sports or exercise per week (0-3 days per week, and 4-7 days per week).

One way ANOVA was used to assess the difference in means of BMI of adolescents by categories of family food behaviors at 14 years and other covariates at 14 years. Logistic regression modelling was used to assess the association of offspring being overweight at 14 years, with significant exposures at 14 years, adjusting for potential confounders. The modelling was done separately for the three categories of family exposures: Psychosocial and behavioural influences and lifestyle factors.

In each series, the association of adolescent BMI (Mean BMI; and two categories, normal weight and overweight/obese) and each exposure was examined separately to identify significant relationships. The variables found to be significant were selected for further analysis. In the first adjusted model, age of the mother (continuous variable) and gender of the adolescent were added as covariates. In the second adjusted model, the other significant variables for this category of influences were also added. In the final model all significant variables across the three categories were included as covariates. SPSS 15.0 software was used for all statistical analysis (SPSS Inc., version 15.0, Chicago, IL, USA).

## Results

Of the original 7223 participants, data on BMI were available for 3862 (53%) adolescent participants (51.9% boys, 48.1% girls) at 14 years FU conducted in 1994-1997. The relationship between family, mother and child characteristics and overweight during adolescence was discussed elsewhere.[23] Participants were mostly white (91%), from middle to higher income (92%) families, their mothers were mostly below 35 years of age (95%) and had completed secondary school or higher education (84%) at the time of birth. The prevalence of adolescent overweight in our study was 25.5% (24.1% boys and 27.3% girls). The mean BMI of the adolescents was 20.6 (±3.8), with males (20.3±3.7) lower than females (21.1±4.0).

The distribution and unadjusted associations between mothers’ reports regarding family meal patterns and influences and mean BMI and overweight of adolescents at 14 years were shown in Table 1.

**Table 1:** Unadjusted associations between family meal patterns and influences and BMI and overweight of adolescents at 14 years^a^

**Family meal patterns and influences**	**Categories**	**Number (%)**	**Mean ** BMI ** (SD) b**	**%Overweight c,d**	**OR of overweight (95% CI)^e^,f**
Frequency of family eating meal together	Once / less han once a week	181 (4.78)	20.6 (3.9)	23.8	1.00
	A few times a week	663 (17.4)	20.7 (3.8)	25.2	1.08 (0.73, 1.59)
	At least once a day	2960 (77.8)	20.6 (3.8)	25.8	1.12 (0.78, 1.59)
	Total/P value g	3804	0.97	0.81	
How important to the mother the family eat together	Very important	1657 (43.6)	20.5 (3.7)	23.3	1.00
	Quite important	1715 (45.1)	20.8 (3.9)	27.2	1.23 (1.05, 1.44)
	Not really important	432 (11.4)	20.9 (4.0)	27.8	1.27 (1.00, 1.61)
	Total/P value	3804	0.01	0.02	
Frequency of family go out to eat	Rarely or never/ several times a year	2314 (60.8)	20.5 (3.7)	23.4	1.00
	Once-twice a month	1059 (27.8)	20.9 (4.0)	27.5	1.17 (1.00, 1.39)
	About once a week	431 (11.3)	21.0 (3.8)	27.8	1.20 (0.95, 1.51)
	Total/P value	3804	<0.01	0.09	
Frequency of having takeaway food	Less than once a month/ rarely or never	2436 (63.3)	20.4 (3.8)	25.6	1.00
	A few times a month	1001 (26.3)	20.5 (3.9)	24.2	0.93 (0.70, 1.22)
	Once or more a week	367 (9.7)	20.7 (3.8)	26.2	1.03 (0.80, 1.32)
	Total/P value	3804	0.10	0.06	
Decision to buy food	I decide completely	1199 (31.5)	20.5 (3.8)	23.6	1.00
	I decide mostly	1605 (42.2)	20.7 (3.8)	25.9	1.13 (0.95, 1.35)
	Partner and I equally/ partner	1000 (26.3)	20.8 (3.9)	27.5	1.23 (1.01, 1.49)
	Total/P value	3804	0.18	0.11	

^a^ For the items in this table mothers were asked how often/important some food behaviours were

^b^ Analysis of variance (ANOVA) used for comparing the mean BMI in each category of variable

^c^ Overweight using BMI and criteria by Cole et al.

^d^ Chi-square used for comparing the percentage of overweight in each category of the variables

^e^ 95% confidence interval (CI); when 1.00 is not included in CI the association is significant

^f^ Logistic regressions used to estimate odds of overweight for each category of the variables

^g^ P≤0.05 was regarded as significant; P=0.05–0.10 was regarded as tending toward significant.

About 78% of the mothers reported eating the family meal at least once a day but only about 44% of the mothers reported that it is very important to them that the family eat together. While slightly less than 40% of the mothers reported that the family goes out to eat about once a week/once-twice a month, about 36% report that the family eats take-away foods once or more a week/a few times a month. In about three quarters of cases, mothers decided which food was bought for the family.

There was no significant association between being overweight at 14 years with mothers’ report on frequency of family eating together and frequency of having take-away foods by the family. Mothers’ report on family attitude towards eating together, frequency of going out to eat and the effect of personal decision to buy foods were found to be significantly associated with overweight at 14 years in this group. These were further examined in the multivariable analysis.

Table 2 shows the results of the multivariable logistic regression analysis. Mothers who reported that family eating their meal together was not important for them were more likely to have overweight children (OR 1.32; CI: 1.03, 1.71) compared to those who stated that family eating was very important to them. Looking across the different models, this association seemed to be independent of family meal patterns and influences, and socio-demographic and lifestyle factors.

**Table 2:** Adjusted odds ratios of association [1] between categories of family meal patterns and influences and adolescents’ overweight at 14 years^a^.

**Family meal patterns and influences**	**Categories**	**Model 1^b^**	**Model 2c**	**Final modeld**
Attitude toward family meal Frequency of family going out to eat	Very important	1.00	1.00	1.00
	Quite important	1.28 (1.08,1.51)	1.28 (1.09, 1.52)	1.28 (1.07, 1.53)
	Not really important	1.32 (1.03,1.71)	1.34 (1.04, 1.74)	1.28 (0.97, 1.68)
	Rarely or never/several times a year	1.00	1.00	1.00
	Once-twice a month	1.13 (0.94,1.34)	1.13 (0.95, 1.35)	1.15 (0.96, 1.40)
	About once a week	1.20 (0.94,1.55)	1.18 (0.92, 1.52)	1.18 (0.91, 1.54)
Decision to buy food	I decide completely	1.00	1.00	1.00
	I decide mostly	1.13 (0.93,1.36)	1.10 (0.91, 1.33)	1.19 (0.98, 1.45)
	Partner and I equally/ partner	1.28 (1.04,1.57)	1.26 (1.03, 1.55)	1.28 (1.03, 1.58)

^a^ Logistic regression analysis used to adjust odds of associations for potential influencing factors

^b^ Adjusted for Gender of adolescent and Age of mother

^c^ Adjusted for Gender of adolescent, Age of mother, Attitude toward eating together, Going out to eat, and Decision to buy food

^d^ Adjusted for gender of adolescent, age of mother, BMI of mother, family income, TV watching, sport, fast food, red meat, soft drink, salad, cooked vegetables, cakes/biscuits, attitude toward eating together, going out to eat, and decision to buy food.

The trend of positive associations between mothers’reports on the frequency of family going out to eat and overweight in adolescents, was non significant in all models. Similarly, the children whose mothers were less involved in selecting food for the family had higher odds of being overweight at 14 years (OR 1.28; CI: 1.04, 1.57) compared to those whose mothers made the primary decisions about buying food most of the time. The association was found to be independent, as no substantial change occurred after adjusting for the range of other potential influences.

The distributions for consumption of specific food items are described in Table 3 . While adolescent’s overweight was significantly associated with frequency of eating cooked vegetables, salads, red meat, soft drink and cakes/biscuits, we did not find statistically significant associations between adolescent’s overweight and frequency of eating sweets, fresh fruits, fast foods and fruit juice.

Table 4 shows the results of multivariable logistic regression with adjustment for variables found to be important in the crude analysis. Apart from the frequency of eating red meat, which lost its significant association after adjustment, other variables did not markedly change their associations with adolescents’ overweight. The positive association between frequency of intake of cooked vegetables and overweight remained significant when adjusted for all of the influencing factors across different models. Similarly, the relatively strong positive association between frequency of drinking soft drink and overweight in adolescents at 14 years was shown to be independent after adjustment.

Table 4 also shows an increase in the consumption of cakes/biscuits that is associated with a decrease in the odds of being overweight among adolescents in the first model. This was significant only for the highest consumers (OR, 0.76; CI: 0.62, 0.97). The trend of the negative association remained the same, but was marginally stronger in the second model when adjusted for other significant family foods consumed (0.65, CI: 0.52, 0.81).

As shown in Table 5, about 62%, 59% and 55% of mothers reported that taste, healthiness and children’s preference of the foods respectively are very important to them when selecting foods. About the same proportion of the mothers reported that fattening of food (37%), cost of food (38%) and partner preference (46%) are very important to them. In contrast, only 24% of the mothers reported convenience to prepare the food as very important to them. Only mother’s report on family concern about food being fattening was found to be significantly associated with adolescents’ overweight.

Table 6 shows the adjusted ORs of association between overweight at age 14 years and “importance of fattening food”, the only family lifestyle factor which showed a significant association in the crude analysis.

The trend in the results shows that an increase in the importance of food being fattening is associated with an increase in the risk of the children being overweight. Furthermore, as can be seen in multivariable modelling, adjusting the results for the range of potential influencing factors makes little difference to the findings.

**Table 3:** Unadjusted associations between frequency of family food consumption and BMI and overweight of adolescents at 14 years^a^.

**Frequency of family food consumption**	**Categories**	**Number (%)**	**Mean (SD)^b^ BMI **	**% Overweight c,d**	**OR of overweight (95% CI)e,f**
Sweets/ lollies	Rarely or never	1672 (45.5)	20.7 (3.8)	25.9	1.00
	2 or 3 times a week	1625 (44.2)	20.6 (3.7)	25.7	1.00 (0.85, 1.16)
	Once or more a day/ most days	381 (9.8)	20.7 (4.4)	24.2	0.91 (0.70, 1.18)
	Total/P value^g^	3678	0.87	0.78	
Fresh fruits	2 or 3 times a week / rarely or never	949 (25.8)	20.7 (3.9)	25.4	1.00
	most days	1180 (32.1)	20.5 (3.8)	24.6	0.95 (0.79, 1.17)
	Once or more a day	1549 (42.1)	20.7 (3.9)	26.7	1.07 (0.89, 1.29)
	Total/P value	3678	0.43	0.43	
Fast foods	Rarely or never	2735 (74.4)	20.6 (3.8)	25.4	1.00
	Once or more a day / most days/2 or 3 times a week	943 (25.6)	20.9 (4.0)	26.6	1.07 (0.91, 1.27)
	Total/P value	3678	0.01	0.43	
Cooked vegetables	2 or 3 times a week / rarely or never	701 (19.1)	20.4 (3.6)	23.4	1.00
	Most days	1728 (47.0)	20.6 (3.9)	24.8	1.08 (0.88, 1.33)
	Once or more a day	1249 (34.0)	20.9 (3.9)	28.1	1.28 (1.03, 1.59)
	Total/P value	3678	<0.01	0.04	
Salad	2 or 3 times a week / rarely or never	1926 (52.4)	20.5 (3.7)	25.3	1.00
	Most days	1130 (30.7)	20.6 (3.9)	25.2	1.00 (0.83, 1.18)
	Once or more a day	622 (16.9)	21.0 (4.0)	27.3	1.10 (0.90, 1.34)
	Total/P value	3678	0.04	0.56	
Red meat	2 or 3 times a week / rarely or never	1807 (49.1)	20.5 (3.7)	24.1	1.00
	Once or more a day / most days	1871 (50.8)	20.8 (4.0)	27.1	1.17 (1.01, 1.36)
	Total/P value	3678	<0.01	0.04	
Soft drink	Rarely or never	1278 (34.8)	20.3 (3.5)	22.0	1.00
	2 or 3 times a week	1246 (33.9)	20.6 (3.9)	24.5	1.15 (0.96, 1.38)
	Once or more a day / most days	1154 (31.4)	21.1 (4.2)	30.9	1.59 (1.32, 1.90)
	Total/P value	3678	<0.01	<0.01	
Fruit juice	Rarely or never	912 (24.8)	20.7 (3.7)	26.0	1.00
	2 or 3 times a week	1116 (30.3)	20.7 (4.0)	25.6	0.98 (0.80, 1.20)
	Once or more a day / most days	1650 (44.9)	20.6 (3.9)	25.6	0.98 (0.81, 1.18)
	Total/P value	3678	0.77	0.97	
Cakes/ Biscuits	Rarely or never	1007 (27.4)	20.8 (4.0)	28.5	1.00
	2 or 3 times a week	1785 (48.5)	20.7 (3.7)	25.3	0.85 (0.71, 1.01)
	Once or more a day / most days	886 (24.1)	20.5 (3.9)	23.4	0.76 (0.62, 0.94)
	Total/P value	3678	0.09	0.03	

^a^ For the items in this table mothers were asked, for each food “How often do you have the following?”

^b^ Analysis of variance (ANOVA) used for comparing the mean BMI in each category of variable

^c^ Overweight using BMI and criteria by Cole et al.

^d^ Chi-square used for comparing the percentage of overweight in each category of the variables

^e^ 95% confidence interval (CI); when 1.00 is not included in CI, the association is significant

^f^ Logistic regressions used to estimate odds of overweight for each category of the variables

^g^ P≤0.05 was regarded as significant; P=0.05–0.10 was regarded as tending to be significant.

**Table 4:** Adjusted odds ratios of association [1] between frequency of family food consumption and adolescents’ overweight at 14 years^a^.

**Frequency of family food consumption**	**Categories**	**Model 1^b^**	**Model 2^c^**	**Final model^d^**
Cooked vegetables	2 or 3 times a week/rarely or never****	1.00	1.00	1.00
	Most days	1.10 (0.88,1.37)	1.11 (0.88,1.40)	1.08 (0.85,1.37)
	Once or more a day	1.29 (1.03,1.61)	1.29 (1.00,1.68)	1.32 (1.01,1.73)
Red meat	2 or 3 times a week/ Rarely or never****	1.00	1.00	1.00
	Once or more a day/most of days	1.23 (1.05,1.43)	1.13 (0.95,1.33)	1.12 (0.94,1.33)
Soft drink	Rarely or never****	1.00	1.00	1.00
	2 or 3 times a week	1.22 (1.00,1.49)	1.26 (1.03,1.54)	1.17 (0.95,1.44)
	Once or more a day/most of days	1.74 (1.43,2.10)	1.86 (1.52,2.27)	1.60 (1.30,1.98)
Cakes/biscuits	Rarely or never	1.00	1.00	1.00
	2 or 3 times a week	0.86 (0.71,1.03)	0.81 (0.67,0.98)	0.83 (0.69,1.02)
	Once or more a day/most of days	0.76 (0.62,0.97)	0.65 (0.52,0.81)	0.71 (0.56,0.90)

^a^ Logistic regression analysis used to adjust results for potential influencing factors

^b^ Adjusted for gender of adolescent and age of mother

^c^ Adjusted for gender of adolescent, age of mother, fast food, red meat, soft drink, salad, cooked vegetables, cakes/biscuits

^d^ Adjusted for gender of adolescent, age of mother, BMI of mother, family income, TV watching, sport, fast food, red meat, soft drink, Salad, cooked vegetables, cakes/biscuits, attitude toward eating together, going out to eat, and decision to buy food.

**Table 5:** Unadjusted associations between influences on family food selection and BMI and overweight of adolescents at 14 years.

**Influences on family food selection**	**Categories**	**Number(%)**	**Mean BMI (SD)^a^**	**Overweight b,c(%)**	**OR of overweight(95% CI)d,e**
Importance of taste of food	Not important/quite important	1342 (38.3)	20.7 (3.9)	25.6	1.00
	Very important	2162 (61.7)	20.6 (3.8)	25.5	0.99 (0.85,1.16)
	Total/*P* value^f^	3504	0.95	0.95	
Importance of children’s preference	Not important/quite important	1581 (45.1)	20.7 (3.9)	25.9	1.00
	Very important	1923 (54.9)	20.7 (3.8)	25.2	0.96 (0.83,1.11)
	Total/*P* value	3504	0.98	0.61	
Importance of partner’s preference	Not important/quite important	1879 (53.6)	20.6 (3.9)	25.5	1.00
	Very important	1625 (46.4)	20.7 (3.8)	25.5	1.00 (0.86,1.16)
	Total/*P* value	3504	0.54	0.96	
Easy to prepare	Not important	730 (20.8)	20.5 (3.9)	23.4	1.00
	Quite important	1915 (54.7)	20.7 (3.8)	26.2	1.16 (0.95,1.41)
	Very important	859 (24.5)	20.8 (4.0)	24.8	1.14 (0.91,1.43)
	Total/*P* value	3504	0.35	0.34	
Healthiness of food	Not important/ quite important	1445 (41.2)	20.7 (3.9)	27.0	1.00
	Very important	2059 (58.8)	20.6 (3.8)	24.6	0.88 (0.76,1.03)
	Total/*P* value	3504	0.24	0.11	
Whether food is fattening	Not important	358 (10.2)	19.9 (3.6)	16.8	1.00
	Quite important	1858 (53.0)	20.7 (3.8)	26.1	1.75 (1.31, 2.36)
	Very important	1288 (36.8)	20.8 (4.0)	27.1	1.85 (1.36, 2.50)
	Total/*P* value	3504	<0.01	<0.01	
Whether food is expensive	Not important/quite important	2164 (61.8)	20.6 (3.8)	24.5	1.00
	Very important	1340 (38.2)	20.8 (4.0)	27.1	1.14 (0.98,1.34)
	Total/*P* value	3504	0.08	0.09	

^a^ Analysis of variance (ANOVA) used for comparing the mean BMI in each category of variable

^b^ Overweight using BMI and criteria by Cole et al.

^c^ Chi-square used for comparing the percentage of overweight in each category of the variables

^d^ 95% confidence interval (CI); when 1.00 is not included in CI the association is significant

^e^ Logistic regressions used to estimate odds of overweight for each category of the variables

^f^ P≤0.05 was regarded as significant; P=0.05–0.10 was regarded as tending to be significant.

**Table 6:** Adjusted odds ratios for association[1] between categories of influences on family food selection and adolescents’ overweight at 14 years^a^.

**Influences on family food selection**	**Categories**	**Model^b^**	**Final modelc**
Whether food is fattening	Not important	1.00	1.00
	Quite important	1.69 (1.24, 2.31)	1.84 (1.33,2.54)
	Very important	1.79 (1.30,2.45)	1.95 (1.40,2.74)

^a^ Logistic regression analysis used to adjust results for potential influential factors

^b^ Adjusted for Gender of adolescent and Age of mother

^c^ Adjusted for gender of adolescent, age of mother, BMI of mother, family income, TV watching, sport, fast food, red meat, soft drink, salad, cooked vegetables, cakes/biscuits, attitude toward eating together, going out to eat, and decision to buy food.

## Discussion

In this cross sectional study of Australian adolescents, reports by the mothers were used as proxies for measures of psychosocial, behavioural and life style influences of family food behaviours on overweight of the adolescents.

We found that aspects of family meal patterns and influences reported by the mothers were associated with overweight amongst the adolescents. While mother’s report on frequency of eating family meal was not significantly associated with overweight, their attitude toward eating together was significantly and inversely associated. Similar findings were observed in the cross-sectional phase of a study conducted in US with adolescent’s aged 9-14 years,where frequency of family dinners was inversely associated overweight, while in the longitudinal phase the association was weakly present in boys only.[28] In three other cross sectional studies, the frequency of family meal was found to be positively associated with consumption of fruits, vegetables, and to some extent, with energy consumption, and negatively associated with consumption of sugar sweetened soft drinks, fried foods and other fatty foods.[24][26][29][30] The differences in the association of mother’s attitude towards eating together on overweight of offspring,compared with frequency of eating together, is possibly due to a broader range of factors influenced by the mothers’ attitudes than is captured by the measures we used, so that an effect of the attitudes on overweight was exerted through factors other than frequency of family eating together.

Another surprising finding in this study was that frequency of the family going out to eat and having take-away food that had no significant association with overweight of adolescents. A number of studies among children have reported that frequency of eating out and having take-away foods have been accompanied by an increase in the consumption of soft drinks and fats, more calories per eating occasion, and with a decrease in intake of fruit, vegetables and low-fat dairy foods.[15][29][31][32] Similar trends have been shown with middle school and high school adolescents and their parents in the US where frequency of fast food purchase was positively associated with consumption of salty and fatty foods.[33] These patterns would generally be expected to predispose towards obesity. Moreover, frequency of food purchased away from home has been shown to be positively associated with BMI of adolescent girls in a small longitudinal study,[34] and percentage of energy coming from foods eaten out of home was reported to be positively associated with BMI among 12-19 years old boys and girls,[35] both studies in the US. Out of three cross sectional studies that specifically examined the association of children’s BMI and fast food or food-away from-home consumption, one study with Iranian adolescents found a positive association in both boys and girls,[36] another study with Mexican children found the association only in girls[37] and the third study conducted in US adolescents found no association.[38] The associations found were relatively weak and no mention was made about adjustment for potential confounders. While some effects of family going out to eat and having take-away food on overweight may be undetected because of study design, the overall set of results also suggests that any relationship may be context specific.

As anticipated, the children whose mothers were more involved in selecting food for the family had lower odds of being overweight at 14 years. This could be a reflection of greater concern of mothers regarding family meals and healthiness of food compared to fathers, which is in line with the association between positive attitudes of the mothers toward family meals with BMI of the offspring discussed earlier. It may also be an effect of bias in reporting, as mothers were the primary source of the data.

Mother’s report on frequency of the family consuming red meat, soft drinks, and cooked vegetables were positively and significantly associated with overweight at 14 years. In contrast, mother’s report on frequency of eating of cakes/biscuits had a significant negative association with overweight. These associations did not change after adjustment for potential confounders except for frequency of eating red meat, where more complete adjustment diminished the significance of the associations. In other studies, percentage body fat was found to be greater among adolescents who had more servings of meat,[15] and frequency of consumption of soft drinks had been associated with overweight.[15][39] In contrast, the finding that frequency of consumption of cakes/biscuits was inversely associated with overweight was unexpected. This may reflect error in reporting of food intake but could also be a true association as increased percentage of energy from carbohydrates has been shown elsewhere to have a protective effect on ovrweight of children.[40] However, it is inconsistent with finding of some small studies regarding a direct association of intake foods high in glycemic index (GI) with children’s overweight, but consistent with findings of some other cross sectional studies regarding relation of consumption of carbohydrates with overweight of children.[29]

Contrary to findings of other studies noted above, frequency of consumption of cooked vegetables by the family was positively associated with overweight of the adolescents. Also we found no association between the frequency of consumption of fruits by the family and overweight of the adolescents. It is possible that preparation of the vegetables (for example adding oil or meats or frying it) caused modification of the protective effects of vegetables, or that overweight individuals in our sample were over-eating all food groups, including vegetables. In addition, as overweight people often tended to over-report more acceptable behaviours (eating more servings of fruit and vegetables in this case), it may reflect a bias in our results. We were not able to distinguish between these explanations in our analysis.

Our findings on the effect of taste, healthiness, child and partner preferences, and cost on selecting food to purchase are similar to those reported in a range of other studies.[41][42] Some have shown that adolescents and parents consumed more fruits, vegetables and low-fat snacks when exposed to low cost fruits, vegetables and low-fat snacks.[43][44][45][46] Similarly, concerns about healthiness of foods and weight status have been found by others to be associated with an increase in consumption of fruits, vegetables and healthy foods and a decrease in consumption of chips and red meat, while convenience to prepare food has been associated with increase in intake of fast foods, chips and unhealthy foods.[41][42] Any such effects in our study were not strong enough to result in an association between these factors and overweight in the adolescents.

The only factor concerned with food purchasing decisions that was associated with overweight was mother’s perceptions about the fattening effect of food, where more importance for this factor had a positive association with overweight. This could reflect more concern about foods causing fattening amongst overweight families and individuals than their normal weight counterparts.

One of the serious concerns in observational studies of dietary intake is underreporting, which can be greater in overweight individuals.[47] We were not able to determine whether under-reporting caused distortion of our results or not. Similar to other studies, although all of the possible measures were taken into account, we cannot be certain that all of the factors that could have an influence on the effect of family food behaviours on overweight of adolescents were controlled. Also as our study is cross-sectional, it is not possible to conclude that the associations represent causal relationships.

Our sample was large and reflected middle to high socio-economic Australian families. One of the main strengths of our study lied in its ability to examine the association of a number of family influences on obesity of adolescents at the same time. The study confirmed the importance of the family and some family food behaviours in shaping risk of being overweight amongst adolescents. Being overweight at 14 years had significant negative associations with mother’s report on the importance of family meals, frequency of eating cake/biscuits, and significant positive associations with mother’s report on frequency of consumption of cooked vegetables, red meats, soft drinks and importance of fattening of foods.

Comparison with the literature suggests that some of these effects may be specific to particular contexts, potentially affected by cultural and socio-economic differences. Longitudinal studies are needed to confirm the findings and to establish the casual relationship between the family influences and the offspring’s overweight.
